# What is the comparative health status and associated risk factors for the Métis? A population-based study in Manitoba, Canada

**DOI:** 10.1186/1471-2458-11-814

**Published:** 2011-10-19

**Authors:** Patricia J Martens, Judith G Bartlett, Heather J Prior, Julianne Sanguins, Charles A Burchill, Elaine MJ Burland, Sheila Carter

**Affiliations:** 1 Manitoba Centre for Health Policy, Department of Community Health Sciences, Faculty of Medicine, University of Manitoba, 408 - 727 McDermot Avenue, Winnipeg, Manitoba, R3E 3P5, Canada; 2 Manitoba Metis Federation Health & Wellness Department, 150 Henry Avenue, Winnipeg, Manitoba, R3B 0J7, Canada; 3 Department of Community Health Sciences, Faculty of Medicine, University of Manitoba, S113 - 750 Bannatyne Avenue, Winnipeg, Manitoba, R3E 0W3, Canada

## Abstract

**Background:**

Métis are descendants of early 17^th ^century relationships between North American Indians and Europeans. This study's objectives were: (1) to compare the health status of the Métis people to all other residents of Manitoba, Canada; and (2) to analyze factors in predicting the likelihood of diabetes and related lower limb amputation.

**Methods:**

Using de-identified administrative databases plus the Métis Population Database housed at the Manitoba Centre for Health Policy, age/sex-adjusted rates of mortality and disease were calculated for Métis (n = 73,016) and all other Manitobans (n = 1,104,672). Diseases included: hypertension, arthritis, diabetes, ischemic heart disease (age 19+); osteoporosis (age 50+); acute myocardial infarction (AMI) and stroke (age 40+); total respiratory morbidity (TRM, all ages). Using logistic regression, predictors of diabetes (2004/05-2006/07) and diabetes-related lower-limb amputations (2002/03-2006/07) were analyzed.

**Results:**

Disease rates were higher for Métis compared to all others: premature mortality before age 75 (4.0 vs. 3.3 per 1000, p < .001); total mortality (9.7 vs. 8.4 per 1000, p < .001); injury mortality (0.58 vs. 0.51 per 1000, p < .03); Potential Years of Life Lost (64.6 vs. 54.6 per 1000, p < .001); all-cause 5-year mortality for people with diabetes (20.8% vs. 18.6%, p < .02); hypertension (27.9% vs. 24.8%, p < .001); arthritis (24.2% vs. 19.9%, p < .001), TRM (13.6% vs. 10.6%, p < .001); diabetes (11.8% vs. 8.8%, p < .001); diabetes-related lower limb amputation (24.1 vs. 16.2 per 1000, p < .001); ischemic heart disease (12.2% vs. 8.7%, p < .001); osteoporosis (12.2% vs. 12.3%, NS), dialysis initiation (0.46% vs. 0.34%, p < .001); AMI (5.4 vs. 4.3 per 1000, p < .001); stroke (3.6 vs. 2.9 per 1000, p < .001). Controlling for geography, age, sex, income, continuity of care and comorbidities, Métis were more likely to have diabetes (aOR = 1.29, 95% CI 1.25-1.34), but not diabetes-related lower limb amputation (aOR = 1.13, 95% CI 0.90-1.40, NS). Continuity of care was associated with decreased risk of amputation both provincially (aOR = 0.71, 95% CI 0.62-0.81) and for Métis alone (aOR = 0.62, 95% CI 0.40-0.96).

**Conclusion:**

Despite universal healthcare, Métis' illness and mortality rates are mostly higher. Although elevated diabetes risk persists for the Métis even after adjusting for sociodemographic, healthcare and comorbidity variables, the risk of amputation for Métis appears more related to healthcare access rather than ethnicity.

## Background

Situated in Canada, Manitoba is a western province with 1.2 million residents, of whom approximately 70,000 self-identify as Métis^a^. The purpose of this study was to determine, using a population-based study, the comparative health status of the Métis people, as well as the risk and protective factors associated with diabetes and for related lower limb amputations.

The Métis are descendants of the early (17^th ^century) economic, social, and political strategic relationships between North American Indians and Europeans [1]. Métis view themselves as distinct from either of their historical ancestors. This is evident in Section 35 of the Canadian Constitution Act of 1982 [2] that states "(1) The existing aboriginal and treaty rights of the aboriginal people of Canada are hereby recognized and affirmed; and (2) In this Act, "aboriginal peoples of Canada" includes the Indian, Inuit and Métis peoples of Canada." Manitoba is considered the homeland of the Métis where they coalesced into a distinct nation in the late 18th century, 'acting collectively' to maintain their homeland, livelihood, and unique culture. Since the 1982 Constitutional recognition of Métis, considerable confusion has remained for many people regarding who is Métis. Such confusion may stem from the differing constructs of '*being of mixed ancestry*', and *'acting as a collective*'. McMillan [3] states "In western and northern Canada [Métis] generally refers to the distinct Métis society which emerged in the nineteenth century, with beginnings along the Red River. Elsewhere, it is often used to designate anyone of mixed Indian-European heritage." The Métis Nation Accord in 1992 defined a Métis as "an aboriginal person who self-identifies as Métis and is a descendant of those Métis who were entitled to land grants or scrip under the provisions of the Manitoba Act of 1870 or the Dominion Lands Act" [3]. To proceed in a collective and self-determining manner on the issue of who is Métis, on September 27, 2002, the Métis National Council adopted a definition: "Métis means a person who self-identifies as Métis, is of historic Métis Nation Ancestry, is distinct from other Aboriginal Peoples and is accepted by the Métis Nation" [4]. The Métis National Council is a body constituted in 1983 by three provincial organizations in Manitoba, Saskatchewan and Alberta [3], and later joined by provincial organizations in Ontario and British Columbia.

There has been a tremendous population growth of Métis in Canada in the past 20 years, with a 43% increase from 1996 to 2001 compared to only a 3.4% increase for all Canadians [5]. In addition to increasing life expectancy and higher birth rates, this may be due to improved enumeration, with a greater number self-reporting Métis identify. However, population-based analyses of Métis health risks in Canada are limited [6], and there may be particular need for information on child health and the underlying social determinants of Métis health [7].

Kinnon [8] commented over a decade ago that people living in poverty experience more chronic health conditions and lower life expectancy, so if Métis are more likely to be in low income groups, one would expect lower life expectancy. Life expectancy for Manitoba Métis people has been reported as 5 to 6 years lower than that of the general population [9-12]. As well, diabetes prevalence is elevated for the Métis compared to the rest of the population, with reports of up to twice the prevalence in Western Canada [10,13-16]. Bruce et al. [17] found the following risk factors for diabetes: sex (Métis females had twice the rate compared to provincial Manitoba counterparts, and males 1.6 times), age, BMI (three-fold increase for those with BMIs of 30 or more), and education (less than Grade 9 education had twice the diabetes prevalence). Income was not associated, but the data came from a relatively low income area (Manitoba Metis Federation's Northwest Region) with the income variable being of a very narrow range [13,14]. After adjusting for age and sex, Métis with diabetes were almost three times as likely to report high blood pressure and heart disease, and twice as likely to report sight impairment compared to Métis without diabetes, and were more likely to report comorbidities [17]. Using 2006 survey data, Janz et al. [16] found that 54% of all Métis aged 15 and over reported having been diagnosed with at least one chronic condition. The most commonly reported chronic health conditions among Métis were arthritis and/or rheumatism (21%), high blood pressure (16%), asthma (14%), and stomach problems or intestinal ulcers (12%); all are similar to the percentages reported in 2001. These rates were higher than those reported in the total population of Canada after age standardizing. For example, almost double the percentage of Métis reported asthma (14% vs. 8%) and diabetes (7% vs. 4%) as compared with the total population.

The objective of this present study was to compare mortality and morbidity rates of Métis people with rates of all others living in the province of Manitoba, Canada, using population-based comparisons based upon data available for all Manitobans. A secondary objective was to analyze the risk or protective factors in predicting the likelihood of having diabetes, and lower limb amputation due to diabetes, to determine if the effect of Métis ethnicity persists even after controlling for various socioeconomic, demographic, health status and healthcare use factors.

## Background on the Manitoba Metis Federation

**Figure 1:**
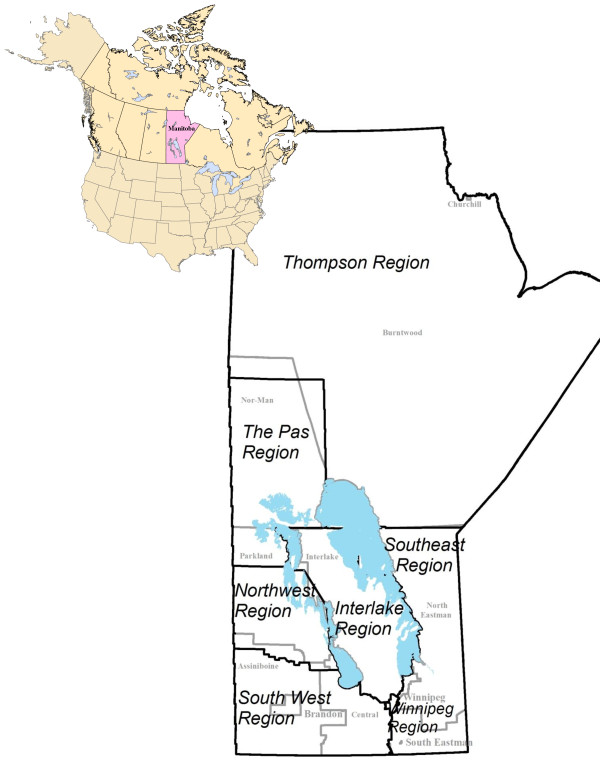
**Location of Manitoba in Canada, and a map of the Manitoba Metis Federation (MMF) Regions of Manitoba (in black), and the Regional Health Authorities (RHAs) (in grey)**.

Founded in 1967 the Manitoba Metis Federation (MMF) is the democratic and self-governing body of the Métis in Manitoba. To be a member of the Manitoba Metis Federation you must self-identify as Métis, show an ancestral connection to the historic Métis community, and be accepted by the contemporary Métis Community^b^. It is important to note that an individual does not need to have two Métis parents in order to meet the criteria for MMF membership--they need only establish their ancestry, connection, and acceptance criteria. For purposes of planning, MMF has seven MMF Regions within Manitoba - Southeast, Interlake, Northwest, Winnipeg, Southwest, The Pas and Thompson. As well, the province of Manitoba has 11 Regional Health Authorities (RHAs). Figure 1 shows MMF Regions and RHAs.

The MMF negotiates with the provincial and federal governments to access funding to provide a wide range of programs and services that are more consistent with Métis cultural norms and responsive to health status differentials. The Manitoba Metis Federation-Health & Wellness Department (MMF-HWD) was created in July 2005 as a Métis-specific 'health knowledge authority' that engages research, policy analysis, program adaptation planning, and community wellness development support in order to contribute to improving Métis health status. Knowing that provincial-level data from such national sample-based surveys as the 2006 Aboriginal Peoples' Survey cannot be used to advise the health system on specific needs by smaller MMF regions, MMF-HWD along with Manitoba Health and the Manitoba Centre for Health Policy (MCHP) decided upon a research project designed to create a Metis Atlas of population-based health status, health care and social services information [18]. MCHP is a research centre in the Department of Community Health Sciences, Faculty of Medicine, of the University of Manitoba. It has a world-wide reputation for its population-based research on health services, population and public health issues.

## Methods

### Study Population and Data

The health status and disease burden of the Métis in Manitoba were compared to all other residents of the province of Manitoba, Canada using a population-based approach. MCHP houses sets of data collectively referred to as the Population Health Services Data Repository. This Repository is an extensive, person-level, linkable yet de-identified Repository of administrative databases for the entire population of Manitoba, covering both health and social services records. Since the provincial Ministry of Health provides comprehensive universal health care coverage for essentially all residents of Manitoba (approximately 1.2 million people), non-participation in the plan is rare and claims data are nearly complete for the population [19,22].

The Repository contains information on mortality and birth, physician and hospital use, pharmaceutical use, use of services such a home care and nursing homes (i.e., long term care facilities) and information derived from education and social services. All of this information is linkable by a scrambled and fictitious unique personal health information number (PHIN). As well, enumeration area information from Canadian Census data, such as neighbourhood-level average household income, can be attributed to the population at an aggregate level via the residential six-digit postal code. This study received permissions from the Faculty of Medicine's Health Research Ethics Board, and from the Health Information Privacy Committee of the Government of Manitoba, as well as from MMF for use of the Métis cohort database.

In order to identify Métis people in the Repository, MMF shared the membership registry with MCHP via the Ministry of Health, who assigned scrambled PHINs to persons on the MMF membership registry through probabilistic linkages on age, sex, and date of birth. As membership in the MMF is voluntary, and Métis members are normally 18 years of age or older, the research team expanded the membership list to include the siblings, children, parents, and grandchildren of the registered MMF member by use of family linkages in the Repository. Other data sources were also used to expand the list: self-identification on Statistics Canada's Canadian Community Health Survey and National Population Health Survey (although these sources only added 1,317 persons to the cohort). The result was a cohort of 90,915 Métis people, 73,016 of which were alive as of December 31, 2006, which closely approximated the self-reported Manitoba Métis population size of 71,805 in the 2006 Census. See Table 1 for the population of Métis and all other Manitobans by RHA, and the population of Métis in each MMF Region.

**Table 1:** Description of the geographic location of Métis and "All Other Manitobans" in 2006

Indicator	Population size of Métis	% of Métis in sub-provincial geographical area	All Other Manitobans	% All Other Manitobans in sub-provincial geographical area
**Manitoba**	73,016		1,104,672	

				

**Regional Health Authorities (RHAs)**				

South Eastman	5,688	7.8	56,390	5.1

Central	4,558	6.2	97,358	8.8

Assiniboine	2,127	2.9	65,909	6.0

Brandon	2,336	3.2	47,185	4.3

Winnipeg	31,647	43.3	633,778	57.4

Interlake	8,817	12.1	67,990	6.2

North Eastman	3,470	4.8	36,809	3.3

Parkland	5,976	8.2	35,986	3.3

Churchill	220	0.3	719	0.1

Nor-Man	4,073	5.6	20,126	1.8

Burntwood	4,104	5.6	42,422	3.8

				

**Manitoba Metis Federation Regions (Metis only)**				

Southeast	9,837	13.5	n/a	n/a

Interlake	8,151	11.2		

Northwest	4,267	5.8		

Winnipeg	31,647	43.3		

Southwest	8,806	12.1		

The Pas	5,974	8.2		

Thompson	4,334	5.9		

For mortality indicators including total mortality, premature mortality (death before the age of 75), mortality due to injury, life expectancy, potential years of life lost and all-cause mortality for individuals with diabetes, deaths were measured over calendar years 2002-2006 using Vital Statistics data, which includes cause of death. Ten years of data, 1997-2006, were required for deaths due to injury to obtain a stable rate.

For prevalence of illnesses, validated algorithms [23] combining diagnoses from hospital admissions and physician visits as well as prescriptions for medications to treat illnesses were applied over five fiscal years, 2002/03-2006/07. Some indicators of prevalence required fewer years of data, in which case the most recent years of data were used up to 2006/07. See Table 2 for the algorithms for each disease measure (diabetes, hypertension, arthritis, ischemic heart disease (IHD), osteoporosis, total respiratory morbidity (TRM), dialysis initiation, acute myocardial infarction (AMI), stroke, and lower limb amputations due to diabetes). TRM is a measure of the burden of all types of respiratory illnesses including asthma, chronic or acute bronchitis, emphysema and chronic airway obstruction. Dialysis initiation is the incidence of individuals in the population who began dialysis treatment during the study period. For AMI and stroke rates, transfers between hospitals were accounted for and only entire hospital episodes were counted to reduce double-counting of events. Rates of lower limb amputations due to complications of diabetes were limited in the denominator to only those individuals with diabetes.

**Table 2:** Definitions of the health status measures, using administrative data available in the Repository housed at MCHP

Health Status indicator	**Definition ***(note: ICD-9-CM is the International Classification of Diseases, 9^th ^Revision, Clinical Modifications; ICD-10-CA is the International Classification of Diseases, 10^th ^Revision, Canada)*
Diabetes prevalence	One or more hospitalizations (1+H) or two or more physician visits (2+P) with a diagnosis of diabetes, ICD-9-CM diagnosis code 250, ICD-10-CA codes E10-E14 (ICD-10-CA used only in hospital abstract data, after April 1, 2004), or one or more prescriptions (1+Rx) to treat diabetes in 3 fiscal years.

Hypertension prevalence	1+H or 1+P with ICD-9-CM codes 401-405, ICD-10-CA codes I10-I13, I15, or 2+Rx in one fiscal year.

Arthritis prevalence	1+H or 2+P with ICD-9-CM codes 274, 446, 710-721, 725-729, 739, ICD-10-CA codes M00-M03, M05-M07, M10-M25, M30-M36, M65-M79, or, 1P and 2+Rx in two fiscal years.

Ischemic Heart Disease (IHD) rate	1+H or 2+P with ICD-9-CM codes 410-414, ICD-10-CA codes I20-I22, 124, I25, or 1P and 2+Rx in five fiscal years.

Osteoporosis prevalence	1+H or 1+P with a diagnosis of osteoporosis, ICD-9-CM code 733 (733.0 only in hospital data), ICD-10-CA code M81, or hip fracture, ICD-9-CM codes 820-821, ICD-10-CA code S72, or spine fracture, ICD-9-CM code 805, ICD-10-CA codes S12.0-S12.2, S12.7, S12.9, S22.0, S22.1, S32.0-S32.2, T08, or humerus fracture, ICD-9-CM code 812, ICD-10-CA codes S42.2-S42.4, or wrist fracture, ICD-9-CM codes 813-814, ICD-10-CA codes S52, S62.0, S62.1, or 1+Rx to treat osteoporosis in three fiscal years.

Total Respiratory Morbidity (TRM) prevalence	1+H or 1+P with ICD-9-CM codes 466, 490-493, 496, ICD-10-CA codes J20, J21, J40-J45.

Dialysis initiation rate	1+P with a physician tariff code for hemodialysis or peritoneal dialysis in five fiscal years

AMI rate	an inpatient hospitalization with a most responsible diagnosis of AMI (ICD-9-CM code 410 and ICD-10-CA code I21) and a length of stay of three or more days unless the patient died in hospital, or a death with AMI listed as the primary cause of death on the Vital Statistics death record.

Stroke rate	an inpatient hospitalization with a most responsible diagnosis of stroke (ICD-9-CM codes 431, 434, 436 and ICD-10-CA codes I61, I63, I64) and a length of stay of one or more days unless the patient died in hospital, or a death with stroke listed as the primary cause of death on the Vital Statistics death record.

Rate of lower limb amputations due to complications of diabetes	an inpatient hospitalization with ICD-9-CM procedure codes 84.10-84.17 or Canadian Classification of Health Interventions (CCI) codes 1.VC.93, 1.VG.93, 1.VQ.93, 1.WA.93, 1.WE.93, 1.WJ.93, 1.WL.93, 1.WM.93. Amputations due to accidental injury (defined by ICD-9-CM codes 895, 896, 897 and ICD-10-CA codes S78, S88, S98, T05.3, T05.4, T05.5, T13.6) were excluded.

### Statistical Methodology

Age- and sex-adjusted rates of mortality and prevalence of illness were calculated using a generalized linear model (GLM) framework for each indicator. Data were analyzed by both the provincial Regional Health Authority (RHA) boundaries, to serve the needs of provincial health planning, and by the seven MMF Regions, to serve MMF socioeconomic program planning (see RHAs and MMF Regions in Figure 1). The adjusted relative risk of death or illness for the Métis in Manitoba compared to all other residents of the province was estimated in Poisson or negative binomial regression models adjusting for age and sex. A variety of age groups or linear and quadratic age terms were employed for various models to ensure the best model fit. Adjusted rates were then obtained by multiplying the estimated relative risk by the appropriate reference rates. Two exceptions are: life expectancy for males and females, which was calculated using a life table methodology based on deaths in the Manitoba population over five calendar years (2002-2006); and potential years of life lost, which was age- and sex-adjusted using direct standardization rather than a GLM, due to the age dependency of the outcome.

For selected indicators, further analyses included explanatory variables beyond age and sex. Logistic regression models were performed for the selected indicators of diabetes prevalence and lower limb amputation rates among people with diabetes, to further explore: (1) differences between Métis and all other residents; and (2) differences among the Métis only. Additional covariates included in the logistic models were: geographical location of residence, average household income of the neighbourhood based on the 2001 Census, major physical illness comorbidity and mental illness comorbidity, and continuity of care. For the model of all Manitobans, aggregate geographical areas were used - the two urban centres of Winnipeg and Brandon, plus an aggregate of North (Nor-Man, Burntwood and Churchill RHAs), Mid (Interlake, Parkland and North Eastman RHAs), and Rural South (Assiniboine, Central and South Eastman RHAs). The model of Métis only used the seven MMF Regions. Comorbidities were measured by the presence of one or more major physical or mental illness Aggregated Diagnostic Groups (ADG) calculated from Johns Hopkins' Adjusted Clinical Group (ACG) case mix system. Major physical ADGs included: ADG 3 = Time Limited: Major, ADG 4 = Time Limited Major-Primary Infections, ADG 9 = Likely to Recur: Progressive, ADG 11 = Chronic Medical: Unstable, ADG 16 = Chronic Specialty: Unstable-Orthopedic, ADD 22 = Injuries/Adverse Effects: Major and ADG 32 = Malignancy. Mental illness ADGs included: ADG 23 = Psychosocial: Time Limited, Minor, ADG 24 = Psychosocial: Recurrent or Persistent, Stable and ADG 25 = Psychosocial: Recurrent or Persistent, Unstable. Continuity of care was measured as the proportion of residents with greater than fifty percent of their ambulatory care visits to the same physician over a two year period. In the logistic regression models, we have 80% power to detect an OR (odds ratio) of 1.038 (or its inverse 0.963) for the diabetes analysis, and 1.279 (or its inverse 0.744) for the amputation due to diabetes analysis. All models had acceptable goodness of fit tests, with a c-statistic ranging from 0.743 to 0.773. All analyses were performed on a Unix server with SAS version 9.1 (SAS Institute Inc, Cary, North Carolina).

## Results

**Figure 2:**
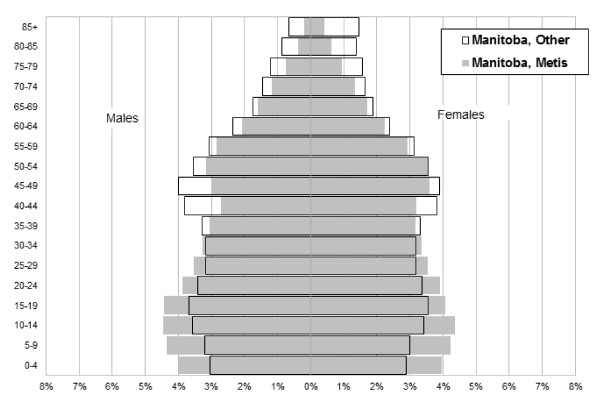
**Age profile (population pyramid) of Métis (n = 73,016) and All Other Manitobans (n = 1,104,672) for 2006, with percentage of males and females in each five-year age grouping**.

The age profile of the Métis compared to all other Manitobans is shown in Figure 2. The Métis have a greater proportion of 0-19 year olds (33.9% vs. 26.4%) compared to all other Manitobans, but a lower portion of older adults aged 65 or more (9.1% versus 13.9%). As well, the highest proportion of both Métis (43.3%) and all other Manitobans (57.4%) reside in the capital city of Winnipeg. A much higher percentage of the Métis live in the Mid and North of the province (36.5% versus 18.5%), which is basically rural, remote or small urban centres (less than 10,000 population, with the exception of the city of Thompson at 13,446 in 2006).

### Mortality and morbidity

The overall health status of Métis people is, for the vast majority of indicators, poorer than that of all other Manitobans. Most measures of mortality, i.e., premature mortality rate (PMR), total mortality rate, injury mortality rate, and potential years of life lost (PYLL), are all statistically significantly higher, at between 14% and 23% higher for the Métis (see Table 3).

**Table 3:** Summary of indicators, comparing rates for Métis and all other Manitobans

Indicator	Provincial difference between Métis and all others (age- and sex-adjusted)	Percentage difference between Métis and all others
*Population health status & mortality*		

Premature Mortality Rate (age 0-74)	4.0 vs. 3.3 per 1000; RR = 1.23 (1.16, 1.30); p < 0.001	23% higher

Total Mortality Rate (all ages)	9.7 vs. 8.4 per 1000; RR = 1.15 (1.07, 1.25); p < 0.001	15% higher

Injury Mortality Rate (all ages)	0.58 vs. 0.51 per 1000; RR = 1.14 (1.01, 1.27); p < 0.03	14% higher

Life Expectancy for Females (from birth) (life table calculation)*	81.0 vs. 81.8 years; RR = 0.99; p = 0.061, NS	NS

Life Expectancy for Males (from birth) (life table calculation)*	75.0 vs. 76.8 years; RR = 0.98; p < 0.001	2% lower

Potential Years of Life Lost (age 1-75) (directly-standardized)*	64.6 vs. 54.6 per 1000; RR = 1.22; p < 0.0001	22% higher

All-Cause 5-year Mortality Rates for Individuals with Diabetes	20.8% vs. 18.6%; RR = 1.12 (1.02, 1.22); p < 0.02	12% higher

*Prevalence of physical illnesses*		

Hypertension (age 19+)	27.9% vs. 24.8%; RR = 1.13 (1.09, 1.16); p < 0.001	13% higher

Arthritis (age 19+)	24.2% vs. 19.9%; RR = 1.22 (1.17, 1.27); p < 0.001	22% higher

Total Respiratory Morbidity (all ages)	13.6% vs. 10.6%; RR = 1.29 (1.19, 1.39); p < 0.001	29% higher

Diabetes (age 19+)	11.8% vs. 8.8%; RR = 1.34 (1.17, 1.51); p < 0.001	34% higher

Rate of Lower Limb Amputations in People with Diabetes (age 19+)	24.1 vs. 16.2 per 1000; RR = 1.49 (1.23, 1.81); p < 0.001	49% higher

Ischemic Heart Disease (age 19+)	12.2% vs. 8.7%; RR = 1.41 (1.31, 1.51); p < 0.001	41% higher

Osteoporosis (age 50+)	12.2% vs. 12.3%; RR = 0.99 (0.89, 1.10); p = 0.826, NS	NS

Dialysis Initiation (age 19+)	0.46% vs. 0.34%; RR = 1.35 (1.17, 1.57); p < 0.001	35% higher

Rate of Acute Myocardial Infarction (age 40+)	5.4 vs. 4.3 per 1000; RR = 1.26 (1.15, 1.39); p < 0.001	26% higher

Rate of Stroke Incidence (age 40+)	3.6 vs. 2.9 per 1000; RR = 1.26 (1.12, 1.41); p < 0.001	26% higher

RR = Relative Rate; 95% CI = 95% Confidence Intervals; NS = not statistically significant

*life expectancy and PYLL were not calculated using regression, hence do not have 95% CIs

Life expectancy is not as sensitive an indicator, and is only 2% lower for Métis males (75.0 versus 76.8 years, p < 0.001), but not statistically significantly different for females (81.0 vs. 81.8 years, p = 0.061; NS). PYLL is 22% higher (64.6 vs. 53.1 years per 1000, p < .0001) for the Métis, as is premature mortality rate (4.0 vs. 3.3 deaths per 1000 before age 75, p < 0.001).

Morbidity is also elevated for the Métis compared to all other Manitobans, with the exception of osteoporosis (see Table 3). The highest disparity occurs for diabetes and related complications - diabetes prevalence is 33% higher for the Métis (11.8% vs. 8.8%, p < 0.001), and lower limb amputation due to diabetes is 49% higher (24.1 vs. 16.2%, p < 0.001). Not surprisingly, kidney dialysis is also elevated, at 35% higher (0.46% vs. 0.34%, p < 0.001). Related cardiovascular diseases, including acute myocardial infarction (AMI) (5.4 vs. 4.3 per 1000, p < 0.001) and stroke (3.6 vs. 2.9 per 1000, p < 0.001) are also more common among the Métis. Manitobans with diabetes have elevated all-cause five-year age- and sex-adjusted mortality rates, but this is even higher in the Métis population compared to all other Manitobans (20.8% vs. 18.6%, p < 0.02). Of note, if the Métis were compared to only the 'all other Manitoba population that do not have aboriginal ancestry, one might expect an even wider mortality and morbidity gap than reported in this study. For example, the poorer health status and reported four-fold differences in diabetes prevalence in First Nations compared to all other Manitobans [24,25] may have been a major contributing factor in the 'all other Manitoba' prevalence of diabetes in this study.

### Regression models of diabetes and lower limb amputation

Tables 4 and 5 show the results of regression modeling that includes variables beyond just age and sex, representing demographics, health status and healthcare use. The first sections of Tables 4 and 5 include all Manitobans by aggregated provincial RHA boundaries, with a comparison of Métis to all others as one of the key variables. The second sections of Tables 4 and 5 show the results of analyses which only include the Métis population, comparing by MMF geographic regions (see Figure 1).

**Table 4:** Logistic Regression Model of Diabetes for all Manitobans, and for Métis only; 2004/05-2006/07 for ages 19+

Covariates	Adjusted Odds Ratio (95% CI)	P-value
**Probability of Diabetes, All Manitobans aged 19+, using aggregate regions**		

Métis (vs. All Others)	**1.293 (1.253, 1.335)**	**<0.001**

Aggregate Regions (ref = Manitoba)		

Rural South	**0.730 (0.717, 0.744)**	**<0.001**

Mid	**0.906 (0.889, 0.923)**	**<0.001**

North	**1.936 (1.888, 1.986)**	**<0.001**

Brandon	**0.882 (0.855, 0.911)**	**<0.001**

Winnipeg	**0.885 (0.872, 0.897)**	**<0.001**

Age, linear	**1.191 (1.187, 1.194)**	**<0.001**

Age, quadratic	**0.999 (0.999, 0.999)**	**<0.001**

Males (vs. Females)	**1.141 (1.123, 1.159)**	**<0.001**

Average Household Income of Neighbourhood (per $10,000)	**0.892 (0.889, 0.896)**	**<0.001**

Mental Illness ADGs	**1.029 (1.008, 1.050)**	**0.0058**

Major Physical Illness ADGs	**1.640 (1.613, 1.667)**	**<0.001**

**Probability of Diabetes, Métis only, aged 19+, using MMF Regions**		

MMF Regions (ref = Manitoba)		

Southeast Region	**0.789 (0.728, 0.855)**	**<0.001**

Interlake Region	**0.849 (0.782, 0.922)**	**<0.001**

Northwest Region	**0.877 (0.785, 0.981)**	**0.0215**

Winnipeg Region	**0.921 (0.872, 0.972)**	**0.0030**

Southwest Region	**0.911 (0.838, 0.991)**	**0.0306**

The Pas Region	**1.219 (1.114, 1.333)**	**<0.001**

Thompson Region	**1.664 (1.488, 1.860)**	**<0.001**

Age, linear	**1.199 (1.184, 1.215)**	**<0.001**

Age, quadratic	**0.999 (0.999, 0.999)**	**<0.001**

Males (vs. Females)	1.003 (0.943, 1.066)	0.9319

Average Household Income of Neighbourhood	**0.875 (0.856, 0.893)**	**<0.001**

Mental Illness ADGs	1.061 (0.983, 1.145)	0.1270

Major Physical Illness ADGs	**1.652 (1.551, 1.761)**	**<0.001**

*95% CI refers to the 95% confidence interval, or confidence limit, of the odds ratio. Bolded OR indicates statistically significant at the p < .05 level or less.

^1^Note: ADGs refers to Aggregated Diagnostic Groups

**Table 5:** Logistic Regression Model of Diabetes-Related Lower Limb Amputation aged 19+ years, 2002/03-2006/07, for all Manitobans and for Métis only

Covariates	Adjusted Odds Ratio (95% CI)*	P-value
**Diabetes-Related Lower Limb Amputation for All Manitobans aged 19+, by aggregate region**		

Métis (vs. All Others)	1.126 (0.904, 1.402)	0.2900

Aggregate Regions (ref = Manitoba)		

Rural South	**0.851 (0.734, 0.986)**	**0.0320**

Mid	**1.219 (1.063, 1.398)**	**0.0046**

North	**1.806 (1.530, 2.131)**	**<0.001**

Brandon	**0.585 (0.429, 0.796)**	**<0.001**

Winnipeg	0.913 (0.813, 1.026)	0.1265

Age, linear	**1.145 (1.106, 1.184)**	**<0.001**

Age, quadratic	**0.999 (0.999, 0.999)**	**<0.001**

Males (vs. Females)	**1.944 (1.711, 2.209)**	**<0.001**

Average Household Income of Neighbourhood (per $10,000)	**0.790 (0.757, 0.823)**	**<0.001**

Continuity of Care	**0.709 (0.624, 0.806)**	**<0.001**

Mental Illness ADGs	0.945 (0.808, 1.106)	0.4834

Major Physical Illness ADGs	**3.251 (2.823, 3.743)**	**<0.001**

**Diabetes-Related Lower Limb Amputation for Métis only, aged 19+, by MMF Region**		

MMF Regions (ref = Manitoba)		

Southeast Region	0.648 (0.327, 1.284)	0.2137

Interlake Region	1.294 (0.781, 2.145)	0.3168

Northwest Region	0.730 (0.328, 1.624)	0.4402

Winnipeg Region	0.997 (0.682, 1.456)	0.9863

Southwest Region	0.947 (0.526, 1.705)	0.8554

The Pas Region	1.282 (0.736, 2.232)	0.3804

Thompson Region	1.351 (0.641, 2.847)	0.4283

Age, linear	**1.212 (1.044, 1.406)**	**0.0115**

Age, quadratic	**0.999 (0.997, 1.000)**	**0.0235**

Males (vs. Females)	**2.362 (1.504, 3.710)**	**<0.001**

Average Household Income of Neighbourhood (per $10,000)	**0.840 (0.713, 0.989)**	**0.0368**

Continuity of Care	**0.618 (0.397, 0.962)**	**0.0330**

Mental Illness ADGs	0.773 (0.430, 1.388)	0.3888

Major Physical Illness ADGs	**2.881 (1.779, 4.665)**	**<0.001**

*95% CI refers to the 95% confidence interval, or confidence limit, of the odds ratio

The regression modeling of the likelihood of having diabetes (see Table 4, All Manitobans section) shows that the elevated diabetes prevalence of the Métis persists (aOR 1.29, 95% CI 1.25-1.34, p < .001) even when controlling for the effects of geography, age, sex, neighbourhood income, and other mental and physical comorbidities. Looking at the predictors of diabetes within the Métis population alone (Table 4, Métis only section), the likelihood of diabetes increases with increasing age (aOR 1.20, p < .001), with a plateauing effect at the older age category, as indicated by the quadratic age variable being below 1. Interestingly, there is no statistically significant difference in prevalence by sex, or by mental illness comorbidity. Neighbourhood income, meaning the average household income of the census enumeration area in which the person resides, shows an inverse relationship, with the higher income areas having lower diabetes prevalence (aOR 0.88, p < .001). There is a high degree of association with comorbid physical illnesses (aOR 1.65, p < .001). The effect of geography is quite profound, with Métis living in the northern MMF regions having the highest likelihood of being diagnosed with diabetes (The Pas Region aOR 1.22, p < .001; Thompson Region aOR 1.66, p < .001). All other MMF regions have rates statistically significantly lower than the overall Métis provincial average. This mirrors the information in Table 4 for all Manitobans, where the North aggregate area shows elevated diabetes prevalence for the entire population of Manitoba, both Métis and others.

Table 5 (all Manitobans section) includes the analysis of the likelihood of one particular adverse outcome of diabetes - lower limb amputation. Comparing Métis to all other Manitobans, there is no statistically significant difference (aOR = 1.13, 95% CI 0.90-1.40, p = 0.29) in the likelihood of Métis having an amputation, once controlled for other effects (geography, age, sex, income, continuity of care, and mental/physical comorbidities). An increased likelihood of amputation is associated with older age (with a plateauing effect at older ages), being male (aOR = 1.94, p < .001), and having major comorbid physical illnesses (aOR 3.25, p < .001), but mental illness comorbidities are not statistically significantly associated. As well, residing in a higher income area, and having continuity of physician care both decrease the likelihood of having a lower limb amputation due to diabetes. When the analysis is limited to Métis only (see Table 5, Métis only section), similar associations are seen, with age, being male and having major physical comorbidities increasing the likelihood of amputation, and the two factors of residing in a higher income area and having continuity of physician care decreasing the likelihood.

Although the likelihood of amputation is statistically significantly higher in certain geographical regions (i.e., living in the Mid or North area of Manitoba) as shown in Table 5 for all Manitobans, this same table shows no statistically significant effects of geography for the Métis only. That being said, a similar trend of elevated likelihood of amputation in the two northern MMF regions (The Pas and Thompson) may indicate type 2 error, i.e., a small sample size not having enough power to detect a statistically significant difference.

## Discussion

### Limitations

One limitation of this study is reliance on the use of administrative claims data alone. Although there have been validation studies completed previously [23], there may potentially be undercounting of diagnoses for those people not seeking medical help. However, that would not be any more likely for those people living in similar geographical locations, so the differences in health status should not be affected. The Canadian universal health care system also equally applies to all, so lack of access through potential income barriers would not be as problematic as in a country without universal health care provisions. A further limitation may be in the establishment of the Métis cohort used in this study. This may have included a small number of people who are not Métis, but who were included through familial relationships using health registry data available to us (for example, if a non-Métis married a Métis, we would have classified both as Métis). If anything, this would most likely reduce the gaps in health status between Métis and all other Manitobans, so that the health inequity found in this study would be maintained or increased if a more accurate identifier were available. That being said, the Métis population count obtained through our method was similar (i.e., less than 2% difference) to that obtained through self-report of the Census.

### Age- and sex-adjusted morbidity and mortality rates

Mortality and morbidity rates are, in general, higher in the Métis population of Manitoba compared to all other Manitobans when we look at age- and sex-adjusted rates. Mortality rates, whether premature mortality, total mortality, injury mortality, or potential years of life lost, all appear to be more sensitive indicators of differences than life expectancy. The former mortality indicators show elevated rates for Métis of 14% to 23%, whereas life expectancy is not significantly different for female Métis, and only 2% lower for male Métis compared to the rest of the population. In terms of morbidity, diabetes (34% higher for Métis) and ischemic heart disease (40% higher) show the biggest gap in a disease outcome for adults aged 19 and older. As well, age- and sex-adjusted diabetes-related outcomes such as lower limb amputations with diabetes comorbidity (49% higher), and dialysis initiation (35% higher) all mirror the elevated diabetes rate for the Métis.

Our research shows higher Métis life expectancies than those reported previously [9-11], and a gap of less than 2 years difference between Métis and all other Manitobans. As well, there appears to be a much smaller gap than found for Manitoba First Nations people, where life expectancy was 8 years lower than the rest of the population [24,25].

In our study, the overall Manitoba age- and sex-adjusted prevalence of diabetes was elevated for Métis compared to all other Manitobans (11.8% vs. 8.8%; Relative Risk [RR] = 1.34). Although the prevalence of diabetes in the Métis population is elevated in Manitoba, this report did not find a doubling or tripling effect as in other Métis studies previously [10,13,14,16]. This may relate to the fact that there may be underlying undiagnosed diabetes, or the rest of the Manitoba population rate is much higher than in some other provinces, or the Manitoba sample in previous studies was different than our population-based cohort that included all Manitoba Métis people. For those living with diabetes, we found an elevated risk of lower limb amputation for the Métis compared to the rest of the population (24.1 vs. 16.2 per 1000) when adjusting only for age and sex. Although a gap exists, in fact, many of the health indicators for the Métis appeared to be somewhat between those rates found for the general population and for the First Nations populations [24-26].

### A more complex analysis of diabetes and amputation - regression modeling to determine factors

The main predictors of diabetes for the Métis were similar to those found in previous research studies, with older age and physical comorbidities both being strongly associated with increased likelihood (see Table 4). The sex difference in the diabetes model was not statistically significant for Métis (aOR = 1.00, 95% CI 0.94-1.07, NS), and only slightly elevated for males in the entire population model (aOR = 1.14, 95% CI 1.12-1.16). This corresponds with the finding of Janz et al. [16], but is contradictory to the finding of Bruce where females had a higher prevalence [13,14].

However, the sex difference for amputation due to diabetes showed a high degree of elevated risk for males for both the entire population model (aOR = 1.94, 95% CI 1.71-2.21) and the Métis only model (aOR = 2.36, 95% CI 1.50-3.71). Although this study did not look at causes of the sex differences, it could be speculated that males have a more rapid progression of disease or may be diagnosed with diabetes at a later stage, thus being more at risk for amputation. Altenberg et al. [27] have demonstrated that people exhibiting lower health-conscious behavior, visiting a healthcare provider less often or showing less anxiety about their diabetes are more likely to have diabetic foot ulcers (which, in turn, could lead to greater risk of amputation). So factors such as these may be more likely in males, hence they would have an elevated risk for amputation - this needs further study in Manitoba to understand the sex differences.

For the Métis only (see Table 4), in contrast to Bruce [13,14], a lower likelihood of having diabetes was associated with higher average household income of the neighbourhood (aOR = 0.875, 95% CI 0.86-0.89 for each $10,000 increase). As well, Métis living in the southern and mid-provincial MMF Regions had less likelihood of diabetes, but Métis living in the two northern MMF regions (The Pas MMF Region aOR = 1.22, 95% CI 1.11-1.33; Thompson MMF Region aOR = 1.66, 95% CI 1.49-1.86) were more likely to have diabetes compared to the provincial average. Bruce et al. [17] did not find a geographical difference, but this may have been a type 2 error due to a much smaller sample size. That study did show a trend towards higher diabetes prevalence in rural/northern compared to urban areas (7.1% vs. 5.7%, NS), albeit not statistically significant. Once again, further study is required to understand why the likelihood of diabetes is higher for Métis living in the North. This is contrary to the finding of Martens et al. [24], where First Nations people had a greater risk of diabetes in the southern Tribal Councils, possibly due to a longer history of colonization, stress and lifestyle changes in more urbanized areas compared to more remote areas.

### Comparing the age- and sex-adjusted models with the more complex models

The regression models of diabetes and of lower limb amputation give a much more complex picture of the risk factors compared to an age- and sex-adjustment only. In Table 4, for all Manitobans, the adjusted odds ratio indicates an elevated likelihood of diabetes for the Métis compared to all other Manitobans (aOR = 1.29, 95% CI 1.25-1.34, p < .001; 29% higher), after adjusting for age, sex, geographical location, income, continuity of care, and comorbidities. This is very similar to the relative risk of 1.34 in Table 3, which is only adjusted for age and sex, showing 34% higher risk of diabetes for Métis compared to all others. Despite controlling for other factors, such as living in the North, in lower income areas, and having more physical comorbidities, there is still the persistent effect of being "Métis" and its association with a higher likelihood of having diabetes. This could possibly be genetic susceptibility of the Métis, due to their First Nations ancestry, or to unmeasured factors in our analysis such as dietary considerations [28] or stress levels [29,30]. Further study would be warranted which includes more comprehensive data sources.

In contrast to the complex modeling of diabetes, the complex modeling of the likelihood of lower limb amputation for those with diabetes shows a dramatically different result when looking at the Métis effect (see Table 5). Table 3 indicates that the age- and sex-adjusted rate of amputation is 49% higher for the Métis compared to all other Manitobans (24.1 vs. 16.2 per 1000, p < .001, RR = 1.49). However, Table 5 shows the more complex regression model with factors such as geography, comorbidity and continuity of care as well as age and sex. This showed no statistically significant difference in the likelihood of lower limb amputation between Métis and all others (aOR = 1.13, 95% CI 0.90-1.40; p = 0.29, NS). Note that the model only has the power to detect an OR of 1.279, so this could be a type 2 error. However, given the dramatic decrease and the small OR after adjustment, even if this were statistically significant it would show that the ethnicity factor of being Métis is clinically not important. So this indicates that factors other than ethnicity have a significant effect on the likelihood of having an amputation once a person has diabetes. Being older or being male, living in an area of lower average household income, or in the Mid and North parts of Manitoba, and having major comorbid physical illnesses all contribute to increased likelihood of amputation. However, having continuity of care shows a beneficial association (aOR = 0.709, p < 0.001). Continuity of care may reflect the difficulties of accessing the same physician for the majority of one's healthcare, especially in rural and northern areas of the province where physician turnover rates may be high. Martens, Bartlett et al. [18] found that provincially, 65.4% of Metis and 69.1% of all other Manitobans received over 50% of their care from the same physician in a two-year period of time of 2005/06 to 2006/07. However, this was much lower in the North area of the province (Métis 58.7%, all other Manitobans 57.2%), and particularly in the RHA of Burntwood (Métis 47.3%, all other Manitobans 47.3%). For diabetes in particular, consistent management and follow-up may prevent adverse outcomes.

So the "Métis" effect of higher amputation rates may be explained by where the Métis live, their lower income levels, their additional burden of comorbidity, or the lower percentage receiving continuity of care. It is important to note, however, that even in the Métis-only analysis, higher average household income level was associated with lower amputation rates, so social policy must be considered. As well, knowing that Métis living in rural and northern remote areas have difficulty accessing the healthcare system, it is important to do further study into healthcare access issues. A previous ecologic study by Martens et al. [26] has shown that diabetes prevalence was highly associated with socioeconomic status amongst First Nations Tribal Council areas of Manitoba, but lower limb amputation was associated with lower rates of access to specialist care (and not with socioeconomic status). This study of the Métis was analyzed at the individual person level, not the aggregate area level, but we see similar findings as to the importance of the healthcare system in being associated with lower rates of amputation for people living with diabetes.

## Conclusions

Despite a universal health care system, the Métis of Manitoba have poorer overall health status as indicated by mortality rates and by a number of physical health conditions. In the case of diabetes, even after adjusting for various competing explanations of poor health such as geographical variation, age, the presence of other physical and mental comorbidities, and average household income, the elevated diabetes prevalence still persists for the Métis. However, for an adverse outcome of diabetes - lower limb amputation - the differential no longer becomes statistically significant between Métis and all other Manitobans. Increased likelihood of having an amputation is associated with being male, being older, having more physical comorbidities, living in a lower income neighbourhood, living in certain parts of the province (notably, the mid and north areas), and having poorer continuity of physician care. Some of these risk factors may be amenable to intervention, such as increasing the continuity of care for those with diabetes (especially in the more rural and northern areas of the province). Further research studies need to examine the potential of reducing the burden of illness through appropriate intervention strategies designed to increase healthcare continuity for the Métis living throughout the province of Manitoba. During production of its comprehensive evaluation, the MMF - Health & Wellness Department interviewed RHA and MMF staff involved in the Department's knowledge translation process. Quotes show that analysis by MMF and RHA geographic boundaries has resulted in joint MMF/RHA ownership of the health challenge that Métis citizens in Manitoba face. Quotes also indicate that such analysis expands the solution discovery process to include areas where planned complementarities between MMF socioeconomic programs and RHA health programs might reduce morbidity and mortality related to diabetes [31].

## Competing interests

The authors declare that they have no competing interests.

## Authors' contributions

PJM and JGB were the co-principal investigators of this study, and conceived of this study. PJM and JGB share joint first authorship on this manuscript. All authors contributed to the details of the design of the study and interpretation of analyses. HJP and CAB performed the statistical analyses. EMJB coordinated the study, and all authors attended meetings for ongoing review and interpretation of the analyses. PJM, JGB, HJP, CAB took the lead on writing the manuscript. All authors read and approved the final manuscript.

## Endnotes

a. Note that in Manitoba, the Manitoba Metis Federation (MMF) uses the term, Metis, without the accent (Métis). This differs throughout Canada. For textual references to MMF and its regions, the accent acute was not used. However, for the sake of less confusion in this text, all other references to Metis will use the accent.

b. Information on the Manitoba Metis Federation can be found at the MMF website: http://www.mmf.mb.ca/

## Pre-publication history

The pre-publication history for this paper can be accessed here:

http://www.biomedcentral.com/1471-2458/11/814/prepub
